# *β*-Catenin Regulation in Sporadic Colorectal Carcinogenesis: Not as Simple as APC

**DOI:** 10.1155/2018/4379673

**Published:** 2018-08-16

**Authors:** Ernst Fredericks, Gill Dealtry, Saartjie Roux

**Affiliations:** Department of Biochemistry and Microbiology, Nelson Mandela Metropolitan University, Port Elizabeth, South Africa

## Abstract

**Background:**

The wnt/APC/*β*-catenin pathway is a critical initiator in colorectal carcinogenesis in both hereditary and sporadic colorectal cancer (CRC). The progression of this process remains incompletely understood, although inflammation is pivotal. Drivers of inflammation are elevated in malignant tissue and have been shown to regulate *β*-catenin expression. Interleukin-17A (IL-17A) is protumorigenic at elevated levels via COX-2 stimulation. Elevated peroxisome proliferator-activated receptor *γ* (PPAR*γ*) expression has reduced risk of carcinogenesis and good overall prognosis in established CRC. Activation of PPAR*γ* has inhibitory effect on *β*-catenin.

**Methods:**

Using qPCR and IHC, we compared *β*-catenin, PPAR*γ*, COX-2, and IL-17A in the colonic mucosa of patients with sporadic CRC, inflammatory bowel disease (IBD), and irritable bowel syndrome (IBS), against a normal control population.

**Results:**

*β*-catenin mRNA and protein expression progressively increased from the Normal group, through IBS and IBD reaching statistical significance in CRC. COX-2 mRNA levels increased similarly with statistical significance in IBD and CRC. However, COX-2 protein expression was inverted with significant expression in the Normal and IBS groups and reduced levels in IBD and CRC. PPAR*γ* mRNA expression was unchanged in IBD and CRC but was significantly elevated in the IBS. IL-17A mRNA was significantly reduced in IBS and CRC but unchanged in IBD. There were no differences in all parameters tested in the Normal and IBS groups.

**Conclusion:**

*β*-catenin is confirmed as a major driver of colorectal carcinogenesis but is controlled by many more players other than APC. Elevated levels of PPAR*γ* may have an anticarcinogenic effect. The role of COX-2 expression, especially its posttranscriptional regulation in colorectal cancer, needs further elucidation.

## 1. Introduction

Sporadic colorectal cancer (CRC) is a heterogeneous disease with multiple factors involved in carcinogenesis. Inherited mutations and spontaneous molecular derangements are important initiators/promoters of the carcinogenic process, but cancer propagation remains unclear. Although mutations have been linked to carcinogenesis, no single gene mutation has been exclusively linked to or identified to be consistently present in all cases of CRC [1]. Therefore, other molecular factors must propagate colorectal carcinogenesis.

The wnt/*β*-catenin signalling pathway, important in the hereditary CRC model, has an equally important role in sporadic colorectal carcinogenesis. Mutations in APC, a tumour suppressor gene, are central in colorectal carcinogenesis through the wnt/*β*-catenin signalling pathway. APC binds *β*-catenin in a complex with glycogen synthase kinase-3*β* (GSK-3*β*) and regulates its activity through phosphorylation and degradation. APC mutation leads to nuclear accumulation of *β*-catenin and transcription of oncogenes resulting in carcinogenesis. Somatic mutations of APC account for 70% of cases of sporadic CRC, while germ line mutations of the same gene are found in 100% of patients with Familial Adenomatous Polyposis [2, 3]. *β*-catenin mutations, resulting in nuclear accumulation of *β*-catenin, have also been noted in a minority of patients with sporadic CRC [4].

In colitis-associated cancer (CAC), an example of inflammatory bowel disease- (IBD-) related CRC, chronic inflammation predisposes to carcinogenesis through signalling pathways involving wnt/*β*-catenin, K-ras, nuclear factor-*κ*B (NF-*κ*B), and downstream cytokines [5]. COX-2, an inducible form of cyclooxygenase, serves as an interface between inflammation and cancer. COX-2 expression and its downstream by-product, prostaglandin E_2_ (PGE_2_), are upregulated in sporadic CRC and IBD [6]. Moreover, COX-2 inhibitors have been shown to reduce carcinogenesis in both animal and human studies [7].

Peroxisome proliferator-activated receptor *γ* (PPAR*γ*), a ligand-activated nuclear transcription factor, has been implicated in colorectal carcinogenesis, although its exact role remains unclear. PPAR*γ* activation is associated with inhibition of cell growth in human colon cancer cell lines as well as cancer xenografts in nude mice. Mice with a heterozygous deletion of PPAR*γ* (PPAR*γ*+/−) have an increased tendency to develop carcinogen-induced CRC [8]. Activated PPAR*γ* inhibits colorectal carcinogenesis through downstream inhibition of the *β*-catenin mediated transcription pathway [9]. Recent data showed that PPAR*γ* stimulation inhibits Th17 activation and IL-17 production [10]. Elevated PPAR*γ* expression confers a good prognosis in CRC [11].

Inflammatory Th17 helper T cells derive their name by their ability to produce IL-17 cytokines (IL-17A-F), among others [12]. Th17 derived cytokines play a role in gut inflammation and carcinogenesis, linking IBD and CRC [13]. Tosolini et al. recently found that increased IL-17A mRNA expression in the tumour tissue is associated with increased cancer recurrence and poor survival, suggesting adverse prognosis [14]. Mechanistically, IL-17 promotes colorectal carcinogenesis through direct stimulation of COX-2/PGE_2_ [15].

These molecules have an intricate interrelationship during carcinogenesis and each one influences the expression and function of the others. COX-2 has been shown to modulate both the wnt/*β*-catenin and PPAR*γ* pathways. PGE_2_ and 15-Deoxy-Prostaglandin J_2_ (15-D-PGJ_2_), downstream products of the COX-2 biosynthetic pathway, increase accumulation and nuclear translocation of *β*-catenin and PPAR*γ*, respectively [16, 17]. PPAR*γ* activation has been shown to inhibit *β*-catenin expression and IL-17 activation [18, 19]. IL-17 has been shown to upregulate COX-2/PGE_2_ [20].

In this study, we examined the interrelationship of *β*-catenin, COX-2, PPAR*γ*, and IL-17 in patients with sporadic colon cancer compared to subjects without cancer. Further comparisons were made in patient populations with irritable bowel syndrome (IBS), with low CRC risk and IBD, with high risk for CRC development [21].

The study results confirm a complex relationship between these role players in colorectal carcinogenesis. In both the Normal and IBS groups, homeostatic mechanisms remain intact with low cell turnover as evidenced by low levels of *β*-catenin and high levels of PPAR*γ*. Inflammation is also kept in check by sufficient COX-2 protein expression and reduced IL-17A expression. These findings were inverted for IBD and CRC.

## 2. Materials and Methods

### 2.1. Study Population

The Human Ethics Committee of the Nelson Mandela Metropolitan University (H07SciBCN-001) approved the study. Written informed consent was obtained from each participant on enrolment. Patients were grouped into (1) Normal: patients attending for screening and surveillance colonoscopy; (2) IBS: patients with constipation- and diarrhoea-predominant IBS using Rome II criteria; (3) IBD: Crohn's disease (CD) and Ulcerative colitis (UC) patients; (4) CRC.

### 2.2. Tissue Collection

Colon mucosal biopsies were taken from the normal caecum and ascending colon in the Normal and IBS groups, from the inflamed mucosa in IBD and from the malignant tissue in CRC. Samples were (i) placed in RNA-later (Sigma) and stored at −20° until analysis and (ii) preserved in 4% paraformaldehyde and embedded in paraffin following standard procedures.

### 2.3. Quantitative Real-Time Polymerase Chain Reaction (qPCR)

Total RNA was extracted (Bio-Rad Aurum™ RNA extraction kit) following the manufacturer's instructions. cDNA was synthesised from 1 *μ*g RNA using the reverse transcription BioRad iScript® cDNA synthesis kit (Bio-Rad Laboratories Inc., USA). The cDNA samples were frozen at −20°C until required.

The GeNorm™ Normalization (Primer Design, UK) algorithm was used to identify three stably expressed reference genes. These were SF3A1 and two Alu repeats, ALUsq and ALUsx [22]. Real-time qPCR reactions were run on an iCycler IQ® system (Bio-Rad Laboratories Inc., USA).

To each well of a 96 well PCR plate, 15 *μ*l of master mix consisting of 10 *μ*l SYBR®Green supermix (Bio-Rad) and 500 nM forward and reverse primers was added. 5 *μ*l of the appropriate cDNA (1:5 diluted) was added to each well except for two wells with no template controls (NTC), where the cDNA sample was replaced with 5 *μ*l of nuclease-free PCR grade water (Ambion, USA). Experiments were run in duplicate.

The protocol for all qPCR runs is comprised of 3-minute Taq polymerase activation at 95°C and 40 cycles of denaturing at 95°C for 30 seconds, 30 seconds at appropriate annealing temperature (Table 1), and extension at 72°C for 30 seconds.

The amplification reaction was followed by a melt curve to verify the specificity of the reaction. The plate was heated to 95°C and then cooled to 1°C below the annealing temperature to ensure that all the DNA was double stranded. The temperature was increased in increments of 0.5°C for 30 seconds up to 95°C to melt the double stranded DNA.

Genes of interest were analysed following the above qPCR protocol and Cq values compared using qBase version 2 software (BioGazelle). Table 1 details all genes used in this study.

### 2.4. Immunohistochemistry

Four micrometer sections were used for IHC staining using the Ventana ES®automated system (Ventana® Medical Systems) following the manufacturer's instructions. The tissue sections were deparaffinised and rehydrated following standard procedures. Antigen retrieval was performed by incubating tissue sections in 10 mM citrate buffer in a microwave for 15 minutes. Slides were transferred to an automated slide stainer. Endogenous peroxidases were quenched by incubating tissue sections with 3% H_2_O_2_ for 30 minutes. Sections were blocked with 10% normal goat serum in phosphate buffer for 10 minutes, then incubated with primary antibody for 32 minutes, washed, and incubated with diluted biotinylated secondary antibody for 6 minutes. The primary antibody for COX-2 was a rabbit monoclonal antihuman antibody and diluted 1:100 in Tris buffer (Ventana®). The secondary antibody was a combined biotinylated goat-anti-mouse IgG and IgM (<200 *μ*g/ml) and biotinylated goat-anti-rabbit IgG (<200 *μ*g/ml) (Ventana®). The primary *β*-catenin antibody was a mouse monoclonal anti-human antibody (0.38 *μ*g/5 ml, Ventana®). The secondary antibody was a combined biotinylated goat-anti-mouse IgG and IgM (<200 *μ*g/ml) and biotinylated goat-anti-rabbit IgG (<200 *μ*g/ml) (Ventana®). Streptavidin horseradish peroxidase (<300 *μ*g/ml) (Ventana®) was applied for 20 minutes. Sections were visualized using 3,3-diaminobenzidine tetrahydrochloride (DAB) (2 g/L Ventana®) as the peroxidase substrate for 5 minutes and Harris's Haematoxylin (standard solution) counterstain. For positive controls, known colorectal cancer slides positive for COX-2 and *β*-catenin were used. Negative control slides were incubated without primary antibody.

All IHC slides were reviewed and scored by an independent pathologist. A scoring system based on the intensity of the reaction and the extent of the staining was used as described by Xiong et al. [23]. Staining intensity was graded as 0 = absent, 1 = weak, 2 = moderate, or 3 = strong. The extent of the positive area was classified as 0 = < 10%, 1 = 10 – 40%, 2 = 40 – 70%, and 3 = > 70%. Slides without stain were considered negative. This method was validated for the immunoexpression of COX-1 and -2 in both UC and CD [24, 25].

### 2.5. Statistical Analysis

All statistical parameters were calculated as mean ± standard error. Statistical comparisons were examined with one-way analysis of variance (ANOVA) followed by the Mann-Whitney U test. The results were considered significantly different at p<0.05.

## 3. Results

A total of 133 patients were recruited. Their demographic details are listed in Table 2.

### 3.1. *β*-Catenin

**Figure 1:**
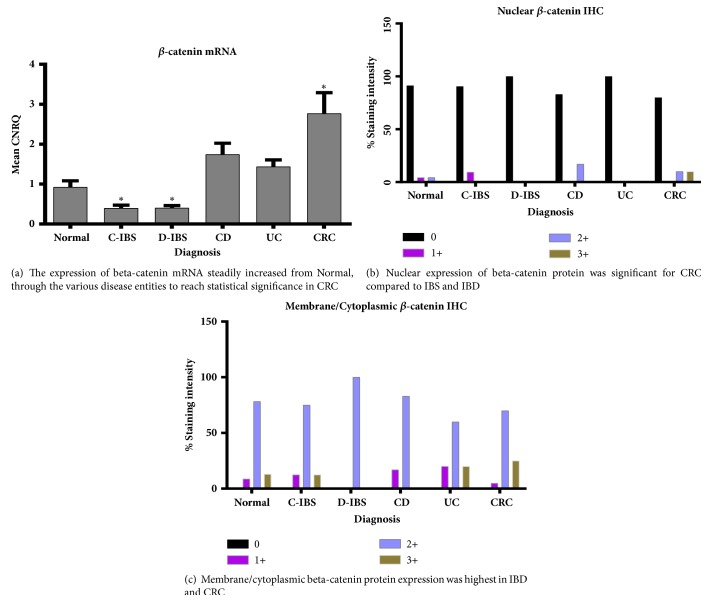
*β*-catenin expression.** (a)***β*-catenin mRNA expression. CNRQ = Calibrated Normalized Relative Quantities, normalized to mean for all samples. Total patient number studied = seventy-five (75) with Normal group: seventeen (17) patients, C-IBS group: twenty-two (22) patients, D-IBS group: eight (8) patients, CD group: six (6) patients, UC group: eight (8) patients, and CRC group: sixteen (16) patients. *∗* denotes significant different versus normal group (p<0.05).** (b)***β*-catenin nuclear expression and** (c)***β*-catenin membrane/cytoplasmic expression by IHC. One-hundred and twenty (120) patient slides analysed with breakdown as follows: Normal group: twenty-five (25) patients, C-IBS: thirty (30) patients, D-IBS: fifteen (15) patients, CD: seven (7) patients, UC: twelve (12) patients, and CRC: thirty-three (33) patients.

**Figure 2:**
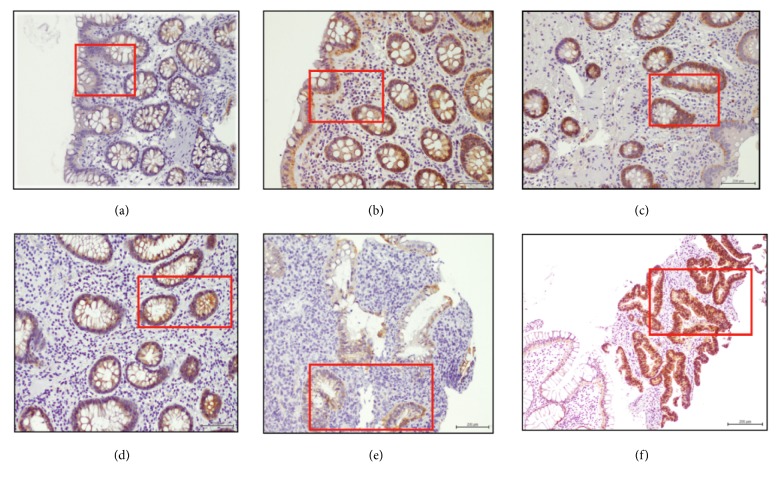
Nuclear and membrane/cytoplasmic *β*-catenin immunoexpression for 122 patients. (a) Normal group (n=25). (b) C-IBS (n=30). (c) D-IBS (n=15). (d) CD (n=7). (e) UC (n=12). (f) CRC (n=33). Significant increase *β*-catenin protein expression from Normal group to CRC, especially nuclear localization. Magnification x40. Scale bar = 200 *μ*m.

*β*-catenin mRNA expression was significantly increased only in CRC (Figure 1(a)). *β*-catenin protein, however, as shown with IHC, showed increased staining at membrane/cytoplasmic level compared with nuclear expression, across all disease groups (Figures 1(b) and 1(c)). Although nuclear *β*-catenin protein expression was low in all groups, CRC had the most expression, being the only group with 3+ scoring. In the IBD group, UC showed no *β*-catenin nuclear expression, while CD had only a small percentage of 2+ scoring. *β*-catenin membrane/cytoplasmic protein expression showed 2+ scoring in all groups, but significant 3+ expression in IBD (UC) and CRC (Figure 1(c)). *β*-catenin membrane/cytoplasmic protein expression generally followed *β*-catenin mRNA expression. *β*-catenin mRNA in the IBS group was significantly lower than the Normal group. *β*-catenin protein expression in C-IBS was similar to the Normal group, while in D-IBS the expression was slightly lower than Normal. This suggests very low cell turnover in these patient groups.  Figure 2 depicts *β*-catenin protein expression.

### 3.2. COX-2

**Figure 3:**
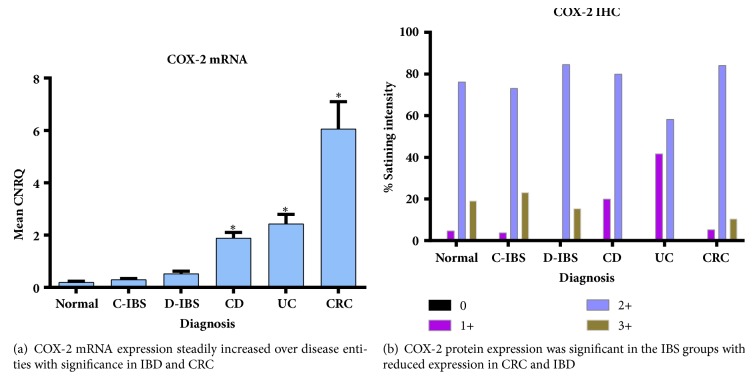
COX-2 expression in the various disease entities.** (a)** COX-2 mRNA expression. CNRQ = Calibrated Normalized Relative Quantities, normalized to mean for all samples. Total patient number studied = seventy-four (74) with Normal group: fifteen (15) patients, C-IBS group: twenty-two (22) patients, D-IBS group: seven (7) patients, CD group: six (6) patients, UC group: eight (8) patients, and CRC group: sixteen (16) patients. *∗* denotes significantly different versus normal group (p<0.05).** (b)** COX-2 IHC. One-hundred and fourteen (114) patient slides analysed with breakdown as follows: Normal group: twenty-six (26) patients, C-IBS: thirty-one (31) patients, D-IBS: thirteen (13) patients, CD: seven (7) patients, UC: twelve (12) patients, and CRC: twenty-five (25) patients.

**Figure 4:**
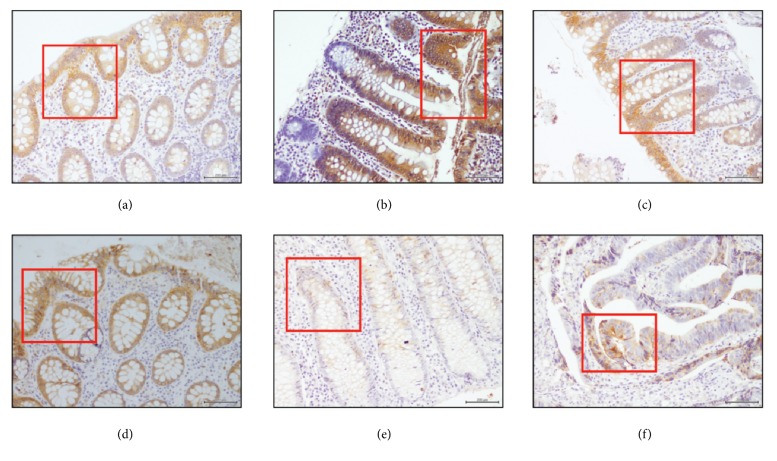
COX-2 immunoexpression in 114 patients. (a) Normal group (n=26). (b) C-IBS (n=31). (c) D-IBS (n=13). (d) CD (n=7). (e) UC (n=12). (f) CRC (n=25). Significant COX-2 protein in Normal and IBD groups with lesser expression in IBD and CRC. Magnification x40. Scale bar = 200 *μ*m.

There was a progressive increase in COX-2 mRNA expression from the noninflammatory groups to the inflammatory groups, with statistically significant increases in IBD and CRC compared to Normal (Figure 3(a)). However, there was an inverse COX-2 protein expression pattern, with more protein expressed in the control and IBS groups and less in the IBD and CRC groups (Figure 3(b)). All groups had COX-2 protein expression, with 2+ scoring of between 60 and 80% of the total COX-2 protein expression. Significant 3+ scoring for COX-2 protein was noted in the Normal and IBS groups compared to no 3+ scoring for IBD and very little 3+ scoring in CRC. COX-2 IHC is depicted in Figure 4.

### 3.3. IL-17A

**Figure 5:**
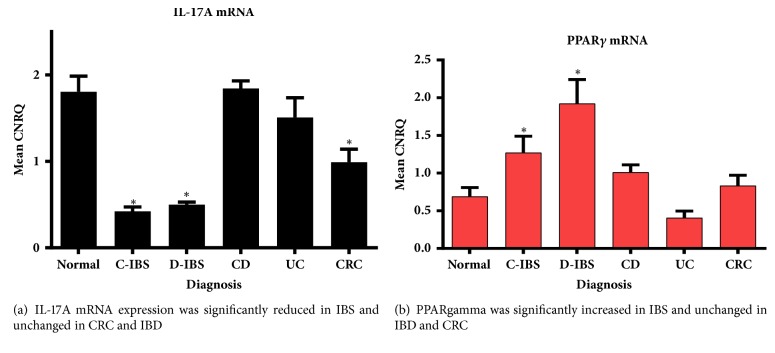
**(a)** Interleukin-17A mRNA. CNRQ = Calibrated Normalized Relative Quantities, normalized to mean of all samples. Total patients studied = 65 with Normal group: thirteen (13) patients, C-IBS group: eighteen (18) patients, D-IBS group: six (6) patients, CD group: six (6) patients, UC group: six (6) patients, and CRC group: sixteen (16) patients.** (b)** PPAR*γ* mRNA expression. Total patient number studied = seventy-four (74) with Normal group: fifteen (15) patients, C-IBS group: twenty-two (22) patients, D-IBS group: eight (8) patients, CD group: six (6) patients, UC group: seven (7) patients, and CRC group: sixteen (16) patients. *∗* denotes significantly different versus normal group (p<0.05).

The expression of IL-17A mRNA was significantly reduced in the non-inflammatory IBS and surprisingly also in CRC (Figure 5(a)). As cytokine expression is generally short-lived with rapid cellular changes, IL-17A protein expression by IHC was not performed.

### 3.4. PPAR*γ*

PPAR*γ* mRNA expression was significantly elevated only in the IBS groups (Figure 5(b)). As PPAR*γ* protein expression was expected to mirror its gene expression and because nuclear localization is transient, IHC was not performed.

## 4. Discussion

Understanding the molecular pathways in carcinogenesis is important to pave the way for novel drug development targeting the signalling pathways involved.  Table 3 summarizes the main findings of the study.

*β*-catenin mRNA and protein expression followed expected trends, especially for IBD and CRC. Serafino et al. similarly found that expression of *β*-catenin protein predominantly had membranous localization in IBD and sporadic CRC [26]. Claessen et al. also noted increased membranous *β*-catenin localization in inflamed IBD mucosa and reduced membranous *β*-catenin staining and increased nuclear *β*-catenin staining in CAC [27]. *β*-catenin mRNA was significantly reduced in IBS in keeping with low cell turnover, while the protein expression was localized to the membrane/cytoplasm. The conclusion from this and other data suggest that *β*-catenin overexpression is a marker of cell proliferation and thus a driver of the carcinogenic process.

COX-2 mRNA is frequently overexpressed in IBD [28] and CRC [29]. Results from this study confirmed significantly increased COX-2 mRNA expression in both these conditions. The protumorigenic effect of COX-2 is thought to be mediated by its downstream product, PGE_2_ [30]. COX-2 protein expression by IHC in our study was reduced in both IBD and CRC, compared to Normal. Similar findings of reduced COX-2 protein expression using IHC and Western blot were shown by Lin et al. in patients with CRC [31]. In contrast, previous investigators showed increased COX-2 protein expression by IHC for both UC and CD, compared to the control group [24, 25]. A limitation of these studies was that no comparison was made with COX-2 mRNA expression. The expression of COX-2 protein in normal tissue is not unusual and has also been shown by both Paiotti et al. and Romero et al. [24, 25]. This is the first report evaluating COX-2 expression in IBS and it showed low COX-2 mRNA expression with relative increased COX-2 protein expression for both C-IBS and D-IBS. A simplistic explanation for this phenomenon may be that a certain level of COX-2 protein expression is required to maintain homeostasis during normalcy, and disruption of this homeostasis with reduced COX-2 protein as in IBD causes the upregulation of COX-2 mRNA expression noted.

Several studies have proven the regulation of COX-2 expression by the wnt/*β*-catenin signalling pathway [32, 33]. Increased *β*-catenin in the cell translocates to the nucleus and stimulates the production of downstream genes,* cyclin D, c-myc, *and* c-jun,* among others that drive cell proliferation and carcinogenesis. In addition, *β*-catenin also binds to HuR, a member of the RNA binding proteins, with subsequent binding to the COX-2 3′UTR to stabilize and regulate the transcription of COX-2 mRNA [34]. However, it is the posttranscriptional regulation of the COX-2 gene, which is far more complex, which controls the expression of the protein.

The role of PPAR*γ* in carcinogenesis remains controversial. PPAR*γ* is highly expressed in normal human colon mucosa as well as human and animal CRC [35]. Ligand activation of PPAR*γ* in colon cancer cell lines is associated with inhibition of cell growth, increased differentiation, and reversal of the malignant phenotype [36]. Despite this, many human colon cancer cell lines are “resistant” to growth inhibition by thiazolidinediones, ligands of PPAR*γ*, and also correlate poorly with levels of PPAR*γ* expression [37, 38]. In our study, expression of PPAR*γ* mRNA in CRC was no different than the Normal group. This suggests that PPAR*γ* seemingly plays no significant role in colorectal carcinogenesis. Similarly, no difference in PPAR*γ* expression was noted for IBD, although there was slight increased expression in CD and slightly reduced expression for UC, compared to Normal. This data is congruent with previous reports showing downregulation of PPAR*γ* expression in UC and unchanged expression from normal in CD [39, 40]. PPAR*γ* was significantly increased for both C-IBS and D-IBS.

PPAR*γ* has important interactions with both *β*-catenin and COX-2. COX-2 activation leads to the production of prostaglandins such as 15-D-PGJ_2_, a natural ligand of PPAR*γ*, with activation of the receptor and downstream signalling. Although not explicitly proven by this study, it is reasonable to extrapolate that high levels of COX-2 protein in IBS could result in increased expression of 15-D-PGJ_2_ and ultimately elevated PPAR*γ* expression. By the same token, low COX-2 protein expression leading to low levels of 15-D-PGJ_2_ may lead to less PPAR*γ* expression or activation, fuelling inflammation and carcinogenesis, as noted in IBD and CRC. As previously discussed, PPAR*γ* directly inhibits *β*-catenin and its nuclear translocation resulting in a nonproliferative state. High levels of PPAR*γ* as seen in the IBS groups may therefore be beneficial. In addition, prostaglandins increase the release of GSK-3*β* from the axin complex, thereby releasing *β*-catenin resulting in nuclear translocation and activation of the wnt/*β*-catenin signalling pathway driving proliferation [41]. Thus, controlling COX-2 expression and its downstream by-products may be an important mechanism to keep PPAR*γ* and *β*-catenin levels in check.

PPAR*γ* also has a direct inhibitory effect on IL-17. IL-17A is a proinflammatory cytokine important in inflammation, autoimmunity, and carcinogenesis. Li et al. showed that IL-17A upregulated COX-2 mRNA and protein in cancer cells lines via NF-*κ*B and ERK1/2 signalling pathways [15]. In CRC, increased IL-17 confers adverse prognosis and poor survival. Xie et al. noted increased IL-17A expression in human colon carcinogenesis [42]. Moreover, IL-17A deficiency was associated with decreased colitis-associated carcinogenesis [43]. IL-17A mRNA was significantly reduced in the CRC group in this study. No clear explanation for this discrepancy can be advanced and the authors suggest a prospective investigation of this in a larger cohort. IL-17A mRNA was significantly reduced in both IBS groups, in keeping with low inflammatory burden. IL-17A mRNA expression in the IBD groups was not different than the Normal group.

To maintain homeostasis, basal expression of these molecules is important for housekeeping, especially to heal inflamed mucosa or to prevent carcinogenesis. Altering the expression of these molecules will disrupt homeostasis, setting the tone for propagation of inflammation (IBD) and CRC. As discussed, COX-2 activation leads to production of PGE_2_ which act via the EP receptors. PGE_2_ upregulation promotes colorectal carcinogenesis by activating the wnt/*β*-catenin pathway through phosphorylation of GSK-3*β* [44]. COX-2 also induces the synthesis of prostaglandin D2 (PGD_2_) and its end product 15-D-PGJ_2_, both of which suppresses inflammation by inhibiting NF-*κ*B and activating of PPAR-*γ* [45, 46]. IL-17A amplifies the COX-2-mediated effects of TNF-*α* and greatly enhances PGE_2_ [47]. PPAR*γ* reduces carcinogenesis by inhibiting both *β*-catenin and IL-17A reducing their downstream signalling and promoting cell differentiation [9, 19].

Results from this study support the complex relationship of the various molecules as documented by other investigators. In conditions with low inflammatory burden, like IBS and Normal groups, COX-2 protein expression is high compared to its mRNA expression. Similarly, PPAR*γ* expression is high with low expression of IL-17A and *β*-catenin. These reported changes in the expression of the investigated molecules appear protective against carcinogenesis. The inverse is true for conditions with high inflammatory burden like IBD and CRC.

The study is not without limitations. The biggest limitation is its snapshot approach with only one time-point measured in a disease course that has linear progression. Future studies should test this hypothesis on animal models of CRC. Posttranscriptional regulation of COX-2 has important implications for CRC and needs elucidation. The potential diagnostic, prognostic, and therapeutic roles of these molecules need investigation prospectively.

## 5. Conclusion

**Figure 6:**
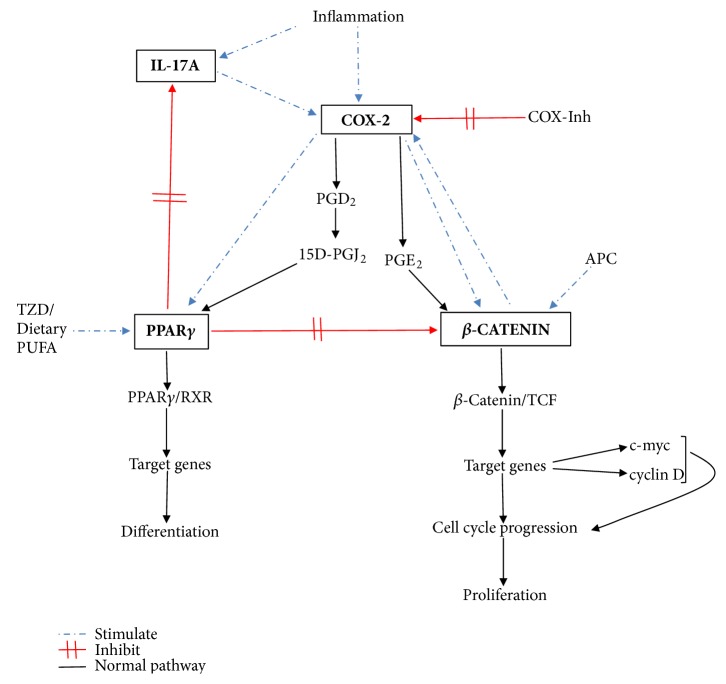
Interaction and regulation between COX-2, *β*-catenin, PPAR*γ*, and IL-17 in colorectal carcinogenesis. TZD: thiazolidinediones, PUFA: poly-unsaturated fatty acids, TCF: T cell factor, RXR: retinoic acid receptor, and Inh: inhibitor.

Although many studies have investigated the role of these molecules in colorectal carcinogenesis, this is the first study comparing them in various disease entities, including IBS, IBD, and CRC.  Figure 6 gives schematic interaction of the molecules controlling proliferation and differentiation. *β*-catenin is confirmed as a major driver of colorectal carcinogenesis, but its expression is controlled by many more players than APC as shown. Although APC mutation may be an important initiator of colorectal carcinogenesis, the propagation of the process is influenced by other molecular factors including COX-2, PPAR*γ*, and IL-17A. Results from this study suggest that mitigating colorectal carcinogenesis would require elevated PPAR*γ* and reduced IL-17A levels. In addition, increased COX-2 protein expression may downregulate *β*-catenin expression. However, longitudinal, prospective studies are required to test this hypothesis and to evaluate gene and protein expression in affected versus unaffected tissue in the same patient.

## Figures and Tables

**Table 1:** Forward and reverse sequences of primers for all genes and their annealing temperatures.

**Gene of interest**	**Forward sequence**	**Reverse sequence**	**Annealing temp (**°**C)**

β**-catenin**	5′-CTGACTTTGCTTGCTTGA-3	5′-CACTATAACTTAACACTACGAG-3′	60

**COX-2**	5′-GAAGCCAATTCAGTAGGT-3′	5′-ACGAAGTGATGAGAAGAC-3′	57

**PPAR**γ	5′-AGGTTTGCTGAATGTGAAG-3′	5′-AATCTGTCTGAGGTCTGTC-3′	60

**IL-17A**	5′-CCACACTCCCCAAAGCAGTT-3′	5′-TGACATGCCATTCCTCAGGG-3′	53

**Reference genes**	**Forward sequence**	**Reverse sequence**	**Annealing temp (**°**C)**

**ALUSq**	5′-CATGGTGAAACCCCGTCTCTA-3	5′-GCCTCAGCCTCCCGAGTAG-3′	60

**ALUSx**	5′-TGGTGAAACCCCGTCTCTACTAA-3′	5′-CCTCAGCCTCCCGAGTAGCT-3′	60

**SF3A1**	Commercial primer, obtained from GeNorm™ Normalization kit. (Primer Design, UK)		60

**Table 2:** Demographic details of study population.

	**Normal**	**C-IBS**	**D-IBS**	**CD**	**UC**	**CRC**
**Total**	26 (19.5)	33 (24.8)	15 (11.3)	8 (6)	14 (10.5)	37 (27.8)

**Age**	53.0	48.0	49.0	43.5	43.5	66.0

**Male**	10 (38.5)	8 (24.2)	5 (33.3)	4 (50)	6 (42.9)	19 (51.4)

**Female**	16 (61.5)	25 (75.8)	10 (66.7)	4 (50)	8 (57.1)	18 (48.6)

**BMI**	25.54	26.14	28.16	21.17	25.96	28.70

**Smoke**	6 (23.1)	7 (21.2)	5 (33.3)	5 (62.5)	1 (7.1)	5 (13.5)

**NSAIDS**	7 (26.9)	3 (9.1)	1 (6.7)	0	0	0

**FH CRC**	1 (3.8)	3 (9.1)	1 (6.7)	0	0	6 (16.2)

( ) =%. Percentage expressed for group in all subcategories, except total.

NSAIDS: nonsteroidal anti-inflammatory drugs.

FH CRC: family history of colorectal cancer.

**Table 3:** The relationship between *β*-catenin, COX-2, PPAR*γ*, and IL-17A.

	*β*-catenin		COX-2		PPAR*γ*	IL-17A
	mRNA	Protein	mRNA	Protein	mRNA	mRNA
IBS	↓↓	↓	↑	↑↑	↑↑	↓↓

IBD	↑	↑	↑↑	↑	*↔*	*↔*

CRC	↑↑	↑↑	↑↑	↑	*↔*	↓

## Data Availability

The data supporting the findings in the study are available from the corresponding author on request.
